# Diffusion Dialysis for Acid Recovery from Acidic Waste Solutions: Anion Exchange Membranes and Technology Integration

**DOI:** 10.3390/membranes10080169

**Published:** 2020-07-29

**Authors:** Chengyi Zhang, Wen Zhang, Yuxin Wang

**Affiliations:** State Key Laboratory of Chemical Engineering, Tianjin Key Laboratory of Membrane Science & Desalination Technology, and School of Chemical Engineering and Technology, Tianjin University, Tianjin 300350, China; zhangqqzhang@tju.edu.cn (C.Z.); yxwang@tju.edu.cn (Y.W.)

**Keywords:** diffusion dialysis, anion exchange membrane, acid recovery

## Abstract

Inorganic acids are commonly used in mining, metallurgical, metal-processing, and nuclear-fuel-reprocessing industries in various processes, such as leaching, etching, electroplating, and metal-refining. Large amounts of spent acidic liquids containing toxic metal ion complexes are produced during these operations, which pose a serious hazard to the living and non-living environment. Developing economic and eco-friendly regeneration approaches to recover acid and valuable metals from these industrial effluents has focused the interest of the research community. Diffusion dialysis (DD) using anion exchange membranes (AEMs) driven by an activity gradient is considered an effective technology with a low energy consumption and little environmental contamination. In addition, the properties of AEMs have an important effect on the DD process. Hence, this paper gives a critical review of the properties of AEMs, including their acid permeability, membrane stability, and acid selectivity during the DD process for acid recovery. Furthermore, the DD processes using AEMs integrated with various technologies, such as pressure, an electric field, or continuous operation are discussed to enhance its potential for industrial applications. Finally, some directions are provided for the further development of AEMs in DD for acid recovery from acidic waste solutions.

## 1. Introduction

**Figure 1:**
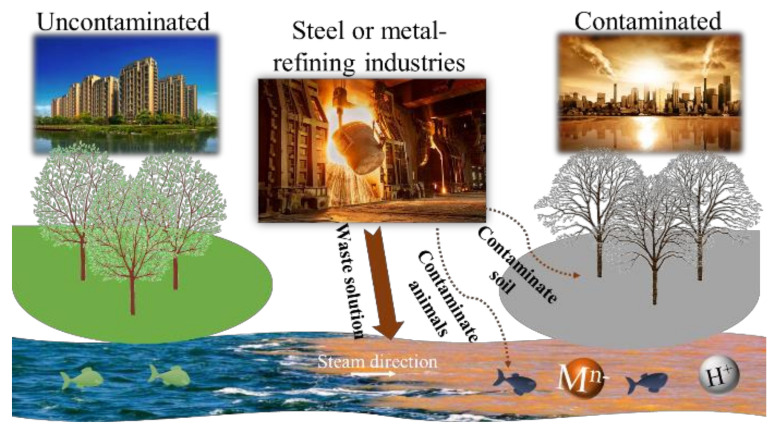
The schematic diagram for the influence from acid waste solutions.

It is well known that large amounts of inorganic acids are widely used in several processes of mining, metallurgical, metal-processing, and nuclear-fuel-reprocessing industries, including pickling, cleaning, leaching, etching, electroplating, and metal-refining and so on. Just for the stainless steel pickling process, it is estimated that at least 0.65 million tons of acidic waste solution is produced in China each year [1]. Dumping this waste into the environment could corrode metal pipes, contaminate the water and soil, and pose severe risks to the health of humans and animals (Figure 1). Accordingly, recovering acid from acidic waste solutions not only saves resources but also protects the environment [2]. Developing efficient and eco-friendly regeneration approaches to recover acid from these industrial effluents has attracted substantial attention and has significant ecological and economic implications.

To date, many methods for recovering acid from acidic waste solutions, such as crystallization [3,4], solvent extraction [5,6,7], ion exchange resin [8,9,10], and membrane technology [11,12,13], have been explored. These methods are summarized including their advantages and disadvantages for acid recovery in Table 1. In the current industrial practices, fluidized bed process or spray roasting is applied to recover HCl from spent pickling solutions. However, the main disadvantage is the high operational cost and high consumption of fresh water and energy [14]. While in some small hot-dip galvanizing plants, precipitation or neutralization process is applied to recover acid. This process is easy and there is no complex installation, but it needs plenty of chemicals and the cost of storage of the sludge is high [15]. Membrane technology is considered to be a simple, effective, and environmentally friendly method to recover acids [16]. This is because the equipment for acid recovery using membrane technology is compact and simple, the effective area of membrane is large and controllable, and there is no by-product produced during the acid recovery process. As one such membrane technology, diffusion dialysis (DD) using anion exchange membranes (AEMs) has been used industrially since 1984 [17]. Compared to other technologies, DD using AEMs for acid recovery has the following significant advantages [16]:

1. Low energy consumption owing to the spontaneity of the process driven by an activity gradient;
2. Low installation costs, simple operation, and maintenance;
3. High product quality due to the high selectivity of AEMs for acids;
4. Environmentally friendliness because of no extra postprocessing and chemical agents.

**Figure 2:**
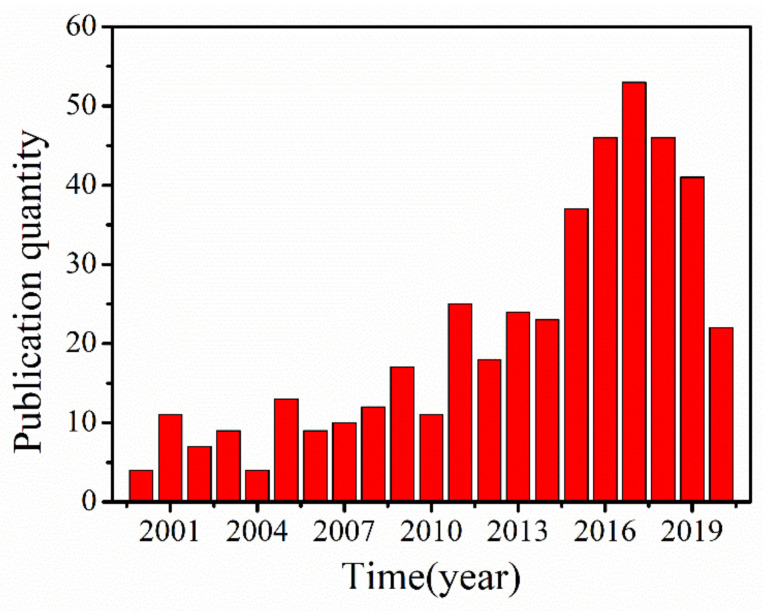
Chronology of diffusion dialysis using anion exchange membranes for acid recovery documents [35].

The DD process was comprehensively applied in acid recovery by researchers, such as in the recovery of inorganic acid (sulfuric acid (H_2_SO_4_) [20], hydrochloric acid (HCl) [21] hydrofluoric acid (HF) [22,23], nitric acid (HNO_3_) [24]) and organic acid (carboxylic acids [25,26]). As the core component, anion exchange membranes (AEMs) which could influence the efficacy of the acid recovery play an important role during the DD process. AEMs as one kind of ion exchange membranes containing positively charge groups allow the migration of anions and repel cations. In fact, AEMs have already attracted much attention in various areas, such as desalination [27], alkaline fuel cells [28,29,30], wastewater treatment [31,32] and so on. At present, some commercial AEMs, including Selemion DSV (Asahi Glass, Tokyo, Japan), Neosepata AFX/N (Tokuyama Co., Tokyo, Japan) and DF120 (Shandong Tianwei Membrane Technology Co., Weifang, China) series are available for recovering acids. However, the acid permeabilities and selectivities of these commercial AEMs are limited [16]. In addition, the economic investment of DD system using AEMs for acid recovery is shown in Table 2. It can be seen that the DD system is high attractive economically. Recently, many efforts to improve the properties of the AEMs used in DD for acid recovery have been made over the years. Figure 2 shows the number of papers published on AEMs for acid recovery via DD from 2000. The number of published papers on this subject is small at the beginning of ten years. However, the number between 2015 and 2019 is approximately double of that between 2009 and 2015, which suggests that the acid recovery via DD began to attract researchers’ attention in the recent five years. This growth could be attributed to the rising environmental regulation and sustainable development issue.

In 2011, Luo published a broad and systematic review of DD for inorganic acid, organic acid and alkali recovery, including the properties of the membranes, nature of the waste solutions and running conditions [16]. In the past decade, many studies on new materials, methods, and technologies for acid recovery via DD were published. Although reviews on acid recovery involving membrane technologies have been published recently, they focused on specific application scenarios, such as acid recovery from acid mine drainage [36,37] and fatty acid recovery [38] or specific technologies, such as electromembrane technology [39] and reactive separation technology [40]. There are few comprehensive reviews that summarize the developments for the key component, AEMs, in detail. Hence, this review intends to bridge this gap and provide an overview of the AEMs for use in DD for acid recovery published in the past decade with a focus on the properties of AEMs, including the acid permeability, acid selectivity, trade-off effect and membrane stability. In addition, to further enhance mass transfer and improve the processing capacity, the integration of DD using AEMs with other technologies for acid recovery is also assessed.

## 2. Description of Acid Recovery Using AEMs

During DD, the ions in an acidic waste solution (the feed solution) migrate through the AEM to the other side, which is filled with deionized water (the receiving solution), driven by an activity gradient. Because of the presence of positively charged functional groups in the AEM, anions (Cl^−^, SO_4_^2−^, NO_3_^−^, etc.) can freely migrate through the membrane, while most cations are blocked by the membrane owing to the Donnan criteria of co-ion rejection [41]. Notably, H^+^, although positively charged, migrates more easily than other cations through the AEM due to its small size, low valence state and high mobility. Therefore, H^+^ can migrate by the dragging effect with the anions from the feed solution into the receiving solution to maintain the electrical neutrality of the solutions [42]. As a result, the acid could be collected on the receiving side, while metal ions could be retained on the feed side. 

**Figure 3:**
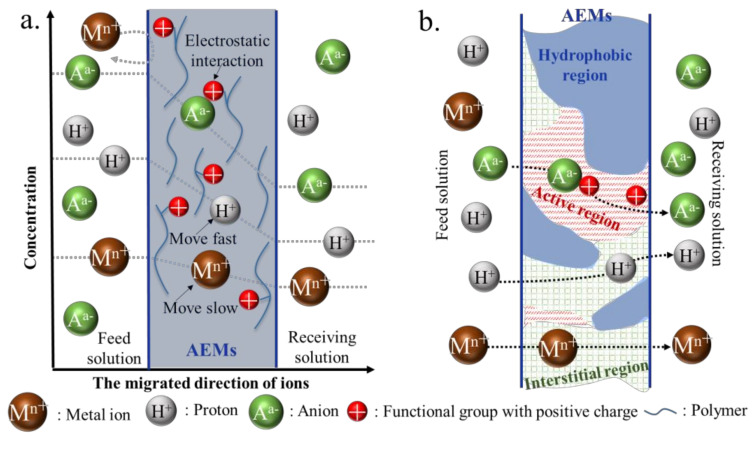
The solution-diffusion model (**a**) and the three-phase membrane model (**b**).

Two models are usually used to describe the migration of acids through AEMs during DD. The first model is the solution-diffusion model, as shown in Figure 3a. The migration of ions through the AEM involves three steps [43]: (1) The ions can interact with the membrane on the feed side via absorption, electrostatic interactions or other interactions. (2) These ions diffuse into the membrane along the activity gradient. (3) These ions divorce the membrane at the receiving side. In the solution-diffusion process, the anions easily migrate through the AEM. However, the crucial step in acid recovery is separating H^+^ and the metal ions. They can be separated over time due to the differences in their solubilities and diffusion rates in the membrane phase during the solution-diffusion process. The second model is the three-phase membrane model, as shown in Figure 3b. During the migration of ions through the AEM, the membrane can be divided into three regions [44,45]: a hydrophobic region, an active region and an interstitial region. The hydrophobic region mainly provides stability and integrity for the membrane. The active region is full of positively charged functional groups for the migration of anions. The interstitial region is considered the swollen region that permits migration of the cations due to the low resistance (such as low electrostatic repulsion) in this area. In this process, the exceptionally high mobility of H^+^ in the interstitial region and anions in the active region via the Grotthus mechanism results in efficient acid recovery.

Here, the dialysis coefficients of H^+^ (*U_H_*) and separation factor (*S*) are used to describe the acid permeability and selectivity of AEMs in the acid recovery process. The *U_H_* could be obtained following the equation,

(1) $$U_{H}=\frac{M}{At\Delta C}$$

where *M* (mol) means the number of ions transported to the permeation solution, *A* (m^2^) is the effective area for dialysis, *t* (h) is the time for dialysis and ∆*C* (mol/L) represents the logarithm average concentration of ions between the two compartments.

The equation of ∆*C* (mol/L) is defined as follows,

(2) $$\Delta C=\frac{C_{f}^{0}-\left(C_{f}^{t}-C_{d}^{t}\right)}{\ln\left[C_{f}^{0}/\left(C_{f}^{t}-C_{d}^{t}\right)\right]}$$

where $C_{f}^{0}$ (mol/L) and $C_{f}^{t}$ (mol/L) mean the concentration of ions in feed solution at initial and selected time, respectively and $C_{d}^{t}$ (mol/L) means the concentration of ions in permeation solution at selected time.

The equation of the separation factor (*S*) is as follow,

(3) $$S=\frac{U_{H}}{U_{M}}$$

## 3. Acid Permeability

Acid permeability is an important factor in acid recovery. Both the kinds of functional groups in the AEM and the structure of the AEM influence its acid permeability.

### 3.1. Alkaline Functional Groups for Permeability

To date, many AEMs based on a variety of polymer materials, including polyether sulfone [46], polysulfone (PSF) [47,48], brominated poly(2,6-dimethyl-1,4-phenylene oxide) (BPPO) [49,50] and polyvinyl alcohol (PVA) [32,44], have been prepared for acid recovery. These polymers do not only serve as the backbone of the membrane but also provide sites for functional group modification of the membrane. Incorporating alkaline functional groups, such as –NR_2_H^+^, –NR_3_^+^, –PR_3_^+^ and so on, into polymer materials is a common method for synthesizing AEMs [51,52,53,54,55]. Overall, the alkaline functional groups, with different types, contents, and substitution sites, have different effects on the acid permeability.

**Figure 4:**
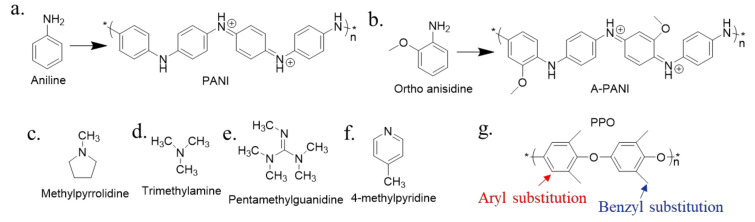
The structure of (**a**) PANI, (**b**) A-PANI, (**c**) Methylpyrrolidine, (**d**) TMA, (**e**) PMG, (**f**) MP, (**g**) PPO [50,56,60,61].

Firstly, the different functional groups have different impacts on the acid permeability. On the one hand, the hydrophilic/hydrophobic characteristics of the membrane could be influenced by different groups, allowing the acid permeability to be tuned. Prajapati [56] prepared two kinds of AEMs using polypropylene (PP) as the substrate: a polyaniline (PANI, Figure 4a)-based AEM and a poly(o-anisidine) (A-PANI, Figure 4b, see Appendix A for the abbreviations of chemicals)-based AEM. The PANI-based AEM was prepared from aniline as the monomer, while the A-PANI-based AEM was prepared from ortho-anisidine as the monomer. The results showed that the acid dialysis coefficient of the A-PANI-based AEM is 42 × 10^−3^ m/h, which was higher than that of the PANI-based AEM (*U_H_* = 32 × 10^−3^ m/h). This is because the A-PANI-based AEM is more hydrophilic due to the higher hydrophilicity of the ortho-anisidine. On the other hand, the different groups have different alkalinity (p*K*_b_), corresponding to different association and dissociation properties with hydroxyl ions, could also influence the acid permeability. Compared to the acid dialysis coefficient of the BPPO-based membrane modified by quaternary ammonium groups using trimethylamine (*U_H_* = 13 × 10^−3^ m/h) [57], that of the BPPO-based membrane modified with pyrrolidinium groups using methylpyrrolidine is 49 × 10^−3^ m/h [50]. The higher anion permeability was a result of the higher alkalinity of methylpyrrolidine (p*K*_b_ = 3.68 at 25 °C, Figure 4c) [58,59] compared to trimethylamine (TMA, p*K*_b_ = 4.20 at 25 °C, Figure 4d) [50], which facilitated the dissociation of anions from the ion exchange groups. Using an alkaline material with a lower p*K*_b_ to modify the membrane might result in a higher acid permeability due to its higher alkalinity. Pentamethylguanidine (PMG, Figure 4e) shows an extraordinarily high p*K*_b_ = 0.2, and Lin [60] used it as a modifier to prepare a guanidinium-based AEM. The obtained AEMs showed excellent hydroxide conductivity. Although it seems that the functional groups with high p*K*_b_ decrease the acid permeability, a different trend is seen when the p*K*_b_ is higher than 7. Khan [61] synthesized BPPO-based AEMs functionalized with 4-methylpyridine (MP, Figure 4f) with a p*K*_b_ of 8.02. Though its p*K*_b_ is much higher than that of methylpyrrolidine, the membranes functionalized with 4-methylpyridine (MP) showed an excellent acid permeability of 66 × 10^−3^ m/h, which is higher than that of the BPPO-based AEM modified with pyrrolidinium mentioned above [50]. This difference might be because MP is weakly acidic which might accelerate the migration of H^+^ [44]. 

Secondly, the degree of functionalization of the AEM can also influence its acid permeability. In Ji’s work [62], both the ion exchange capacity and the water uptake increased with increasing the degree of quaternization, which improved the acid permeability. This phenomenon was also observed in acid recovery using the PVA-based membranes modified by multisilicon copolymers by Wu [63]. The results showed that the acid permeability increased from 10 × 10^−3^ to 29 × 10^−3^ m/h with the increasing content of multisilicon copolymer.

Thirdly, the different sites of functionalization have different influences on acid permeability. Xu [57] found that the H_2_SO_4_ recovery rate using poly(2,6-dimethyl-1,4-phenylene oxide) (PPO)-based AEMs increased with increasing benzyl substitution but not aryl substitution, as shown in Figure 4g. 

### 3.2. Acid-Alkali Functional Groups for Permeability

Considering the advantages of functional groups with weak acidity, polymer backbones were prepared with both alkaline and acidic functional groups to form acid-base ion pairs, which may facilitate acid permeability.

On the one hand, the acidic functional groups could improve the migration of H^+^ in the AEM due to electrostatic attractions [64,65]. Irfan [64] incorporated both quaternary nitrogen and –COOH groups into PPO to recover acid. In the membrane, the quaternary nitrogen permitted the migration of Cl^−^, while the –COOH groups provided sites for the migration of H^+^. The quaternary nitrogen allowed H^+^ to migrate passively, while the –COOH groups allowed active H^+^ migration. As a result, the acid permeability of this membrane was 19 × 10^−3^ m/h, which is slightly higher than that of quaternized PPO (*U_H_* = 13 × 10^−3^ m/h). In addition, a semi-interpenetrating network-based AEM was synthesized by Cheng [65] from polyvinyl chloride (PVC), dimethylaminoethyl methacrylate (DMAM) and divinylbenzene (DVB) via polymerization and quaternization for HCl recovery. The presence of –COOH groups from DMAM could accelerate the migration of H^+^, and as a result, the membrane exhibited high acid permeability (*U_H_* = 40 × 10^−3^ m/h).

On the other hand, the acid-alkali ion pair could form a hydrogen bonding network that enhances the migration of H^+^. Polyvinyl alcohol (PVA) has been widely used as a polymer backbone to prepare AEMs with enhanced acid permeability due to its high content of –OH groups [66]. Mondal [32] prepared a PVA-based AEM mixed with quaternary aromatic amine groups from quaternary 4,4′-(1,1′-biphenyl-4,4′-diyldioxy)dianiline (QBAPB) for HCl recovery via DD. The presence of -OH groups in QBAPB and PVA allowed the formation of a hydrogen-bonded network, which accelerated the migration of H^+^. Emmanuel [67] synthesized a PVA-based membrane modified with 1,4-diazabicyclo [2,2,2]octane (DABCO). The results showed that the membrane had good acid permeability (*U_H_* = 45 × 10^−3^ m/h). The hydrogen-bonded network constructed from the –OH groups of PVA and the N atoms of DABCO also enhanced the migration of H^+^. In addition, Yadav [68] prepared a PSF-based membrane by incorporating neem leaves powder (NP) for acid recovery. The NP contained many functional groups, such as –COOH and –OH. These groups could form a hydrogen bond network, facilitating the migration of H^+^.

### 3.3. Membrane Structure

Considering the low ion migration resistance due to the presence of gaps in the membrane, preparing porous membranes is one strategy for improving acid permeability [69]. The pores can provide channels for ion migration with less resistance. Recently, in DD for acid recovery, porous membranes have also attracted much attention. 

The first porous membrane is pore-filled AEMs, which are prepared by soaking a porous substrate into the monomer mixture followed by a radical polymerization process. As a result, a guest polyelectrolyte gel is introduced into the pores of a host polymer [70,71,72]. Chava [73] prepared a pore-filled AEM by filling the microporous substrate of polypropylene (PP) with organosiloxane-based organic-inorganic hybrid anion exchange microgels. The membrane showed a higher HNO_3_ permeability compared to the commercial Selemion membrane, which is a dense and aminated polysulfone membrane. This is because the PP-based pore-filled membrane had thin and dense layers at surfaces and a porous interior, which was effective for acid diffusion. In addition, Kim [74] prepared a pore-filled AEM using porous polyethylene (PE) as the substrate for H_2_SO_4_ recovery from the FeCl_3_–H_2_SO_4_ solution. The acid permeability of this pore-filled AEM is almost triple as high as that of the dense membrane Neosepta-AFX which is aminated polystyrene-co-divinylbenzene.

**Figure 5:**
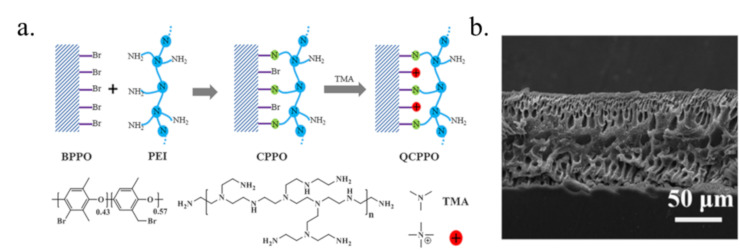
The preparation of the BPPO-based ultrafiltration membrane after modification and amination (**a**) and the cross section from SEM of the BPPO-based ultrafiltration membrane after modification and amination for 2 h (**b**) [77].

The second porous membrane is an ultrafiltration membrane prepared by the phase-inversion method containing a thin surface layer and a microporous supporting substrate [75]. The polymers commonly used as substrates to prepare ultrafiltration membranes are PPO or PSF [47,76]. Lin [77] prepared a PPO-based ultrafiltration AEM modified with polyethyleneimine (PEI) and aminated with trimethylamine (TMA) shown in Figure 5a. There are plenty of pores which do not penetrate through the whole membrane but could accelerate the migration of acid (Figure 5b). Compared to the commercial DF-120 membrane, the optimal BPPO-based ultrafiltration membrane after modification and amination exhibited 6.4 times higher HCl permeability. Similarly, Sun and his coworkers [78] also prepared a PPO-based ultrafiltration AEM that contained –COOH and quaternary ammonium groups. Compared to the acid permeability of the dense PPO-based membrane bearing both quaternary nitrogen and –COOH groups (*U_H_* = 5–19 × 10^−3^ m/h) [64], this porous membrane exhibited a higher acid permeability (*U_H_* = 20–25 × 10^−3^ m/h). Asymmetrically porous AEMs based on PSF and modified with N,N,N′,N′-tetramethyl-1,3-propanediamine (TMPDA) were prepared by Lin [47]. The membranes showed excellent acid permeability of 65 × 10^−3^ m/h, which was 6.6 times higher than that of the commercial DF-120 membrane. This was because this membrane possessed a supporting layer with an appropriate number of pores and a selective layer thickness of 0.5–0.6 μm, which was notably thinner than that of conventional compact membranes, resulting in reduced resistance in the migration of H^+^. In addition, Jyothi [79] used the phase-inversion process to prepare a PSF-based porous membrane by incorporating different contents of eggshell membrane (ESM) power to recover HCl from FeCl_2_–HCl solutions. The acid permeability of this membrane was approximately 5.55 times higher than that of the commercial DF-120 membrane due to the porous structures.

The third porous membrane is a kind of nanofiber AEM prepared by electrospinning and posttreatment. Pan [80] prepared novel nanofiber AEMs from a quaternized PPO/silicon dioxide hybrid material. The acid permeability of the nanofiber membrane prepared by electrospinning and posttreatment is 1.3 times that of the membrane prepared by the conventional casting method. The loose microscale structure of the membrane could facilitate acid permeability better than that of the compact membrane.

## 4. Acid Selectivity

Excellent acid selectivity is a key factor for collecting high-quality acid products during DD so that they can be reused in the steel or metal refining industry. The type of functional groups and the size-sieving effect have main influence on improving acid selectivity in the DD process.

### 4.1. Alkaline Functional Groups for Selectivity

The functional groups in the AEMs not only affect the acid permeability mentioned in Section 3.1 but also influence the acid selectivity during DD.

According to the mechanism of acid recovery using AEMs in the DD process, alkaline functional groups can allow anion migration and block cations due to their positive charge. However, the electrostatic repulsion between the groups and H^+^ is lower than that between the groups and metal ions owing to the small size, low valence state and high mobility of H^+^ [81]. Hence, H^+^ and metal ions can be effectively separated during DD.

Firstly, different alkaline functional groups show different electrostatic repulsions to cations, resulting in different acid selectivities. Pyridinium was used to modify the PVA-based AEM for HCl recovery from FeCl_2_–HCl solutions [82]. In addition, Emmanuel prepared an imidazolium functionalized PVA-based AEM for acid recovery [83]. Compared to the acid selectivity of the PVA-based membrane functionalized by quaternary ammonium groups (*S* = 21) [84], both the PVA-based AEM modified with pyridinium (*S* = 58) and the PVA-based AEM modified with imidazolium (*S* = 53) exhibited higher acid selectivities. Khan [61] also prepared a pyridinium functionalized PPO-based AEM to recover acid. The membrane showed an excellent acid selectivity of 78, which was higher than that of the commercial DF-120B membrane (*S* = 24), which is a quaternized PPO-based membrane with polyester as the substrate [49]. Pyridinium and imidazolium are promising functionalized materials for AEMs to improve acid selectivity during DD. In addition, Pyrrole has an excellent affinity for anions and repulsion for cations [74], and therefore the AEM modified by pyrrole might show a good acid selectivity. Kim [85] used pyrrole to modify surfaces of the commercial AEM, Neosepta-AFX. The acid selectivity of Neosepta-AFX modified by 5 vol.% pyrrole was almost twice higher than the original Neosepta-AFX.

Secondly, the density of the functional groups in the membrane can influence the acid selectivity. Cheng synthesized a series of PVA-based AEMs by grafting different contents of allyltrimethylammonium chloride with a large number of quaternary ammonium groups to recover HCl from FeCl_2_–HCl solutions [66]. The grafting ratio (GR) was correlated with the content of quaternary ammonium groups. The results showed that the acid selectivity improved with increasing GR in the range of 8–26%. A higher content of quaternary ammonium groups in the membrane resulted in stronger electrostatic repulsion of cations. Electrostatic repulsion has a greater influence on Fe^2+^ migration than on H^+^ migration. Hence, higher acid selectivity (*S* = 23) was obtained at a higher GR (26%).

**Figure 6:**
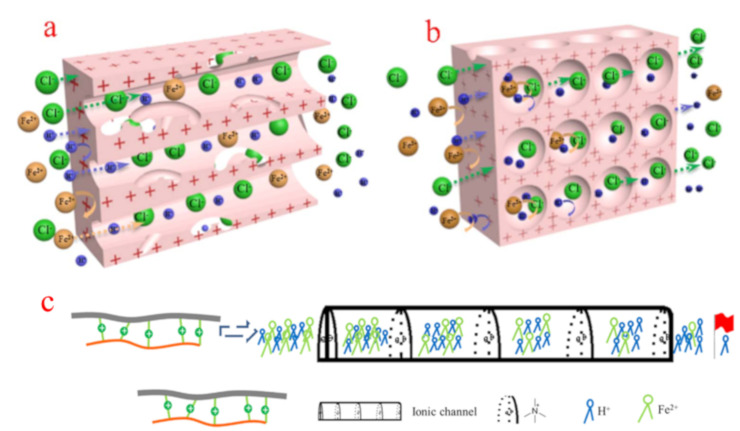
Plausible physical model of ions transferred in the finger-like (**a**) sponge-like structures during the DD process (**b**) and the separation of H^+^ and Fe^2+^ through the quaternary ammonium groups-based ionic channel (**c**) [86,87].

Thirdly, the morphology of the AEM could also influence the acid selectivity. Ge and coworkers [86] prepared PPO-based porous membranes with different pore structures functionalized with quaternary ammonium groups. The acid selectivity for the AEM with a sponge-like pore structure is eight times higher than that of the membrane with finger-like pore structures. Compared to the AEM with finger-like pores, the sponge-like pores were disconnected from each other in the AEM, leading to the formation of a multilayered barricade in the migration path of ions (Figure 6a,b). Ions need to pass through more functional groups in the AEM with sponge-like pores. Hence, the AEM with a sponge-like structure will possess better acid selectivity.

Finally, the position and arrangement of the functional groups can influence the acid selectivity. Xu [22,57] found that the acid selectivity increases to a greater extent with aryl substitution by quaternary ammonium groups than benzyl substitution with the same groups in the PPO-based membrane, as shown in Figure 4g. In addition, Ge [87] prepared a PPO-based AEM functionalized with quaternary ammonium groups arranged in a more linear manner. Briefly, the BPPO was crosslinked by multiamine oligomers, forming an ionic column area full of quaternary ammonium groups between the BPPO and the multiamine oligomer (Figure 6c). As a result, the high acid selectivity for an AEM was obtained (approximately *S* = 2074), which is dramatically higher than that of commercial AEMs. Every quaternary ammonium group is an exclusion site, and the linearly arranged quaternary ammonium groups form a connective ionic channel to continuously separate H^+^ and metal ions.

### 4.2. Acid-Alkali Functional Groups for Selectivity

Incorporating both acid and alkaline functional groups into the polymer backbones can also be used to enhance acid selectivity [88]. Polyelectrolyte complexes (PECs)/PVA membranes containing –N^+^(CH_3_)_3_ and –SO_3_^−^ were prepared by Wang [44] for HCl recovery. On the one hand, the hydrogen bonding network from the –OH of the PVA could facilitate the migration of H^+^ better than the metal ions. On the other hand, the presence of acid groups in the membrane may lead to a charge neutralization microphase, which could improve the migration of cations with low resistance. However, the promotion of the migration of metal ions might be less obvious due to their larger size and higher valence compared to H^+^. The results showed that the acid selectivity of this PECs/PVA membrane was in the range of 57–90, which was higher than that of the membrane without –SO_3_^−^ (*S* = 40).

### 4.3. Size-Sieving Effect

The size-sieving effect is based on the different sizes of the metal ions and H^+^. If the sizes of the pores in the membrane are between the sizes of the metal ions and H^+^, H^+^ can migrate through the pores, while the larger metal ions might be blocked by these pores. Sun [89] used the sieving effect of graphene oxide (GO) nanocapillaries to separate Fe^3+^ and H^+^. The GO membrane showed a layered structure that could block ions with hydrated radius larger than 4.5 Å. Therefore, Fe^3+^, which has a hydrated radius of 4.57 Å, could be confined by the GO membrane, while H^+^ could migrate via the hydrogen bonding network in the GO membrane with less resistance. As a result, the migration rate of H^+^ was two orders of magnitude larger than that of Fe^3+^. 

Obviously, the size-sieving effect influences the separation of H^+^ and the metal ions and is applied in many fields, such as the separation of monovalent and multivalent ions [90], desalination [91], and gas separation [92]. However, applications of the size-sieving effect in acid recovery are limited, and further investigations could focus on relevant materials, including carbon nitride, layered double hydroxide, metal organic frameworks, and covalent organic frameworks.

The acid permeabilities and selectivities for H^+^ over metal ions of the reported membranes are listed in Table 3. The porous membranes and the AEMs with acid-alkali functional groups exhibited a higher acid permeabilities (such as the BPPO-based AEM modified by PEI and TMA [77] and the PSF-based AEM [47]) or selectivities (such as the PPO-based AEM with quaternary nitrogen and –COOH groups [64] and the PECs/PVA AEM [44]). However, Table 3 shows that many membranes show a trade-off effect, which means that membranes with excellent acid permeability exhibit poor acid selectivity and vice versa. Hence, solving these problems will require further study.

## 5. Trade-Off Effects between Acid Permeability and Selectivity

Though many approaches were used to improve the properties of AEMs for acid recovery during DD, there are trade-off effects between these properties, which limits the application of these AEMs in industry [93]. The common trade-off effect is between acid permeability and selectivity [94]. Ji [62] prepared a series of PPO-based AEMs with quaternary tris(2-(2-methoxyethoxy)ethyl)amine (TDA). The water uptake of the membranes increased with increasing ion exchange capacity. As a result, these AEMs exhibited dialysis coefficients of H^+^ ranging from 2 × 10^−3^ to 60 × 10^−3^ m/h with increasing TDA in the membranes, while the acid selectivity dropped from 1682 to 19. It showed an extreme imbalance between acid permeability and selectivity. The same trade-off effect is also seen in porous AEMs where the pores could improve the acid permeability at the expense of the acid selectivity [77]. 

As mentioned above, the incorporation of acid functional groups could enhance both the acid permeability and selectivity, as stated in Parts 3.2 and 4.2, due to the formation of hydrogen bonding networks for the migration of H^+^. In addition, acid functional groups, such as carboxylic acids, have a stronger affinity for high valent metal ions via electrostatic attractions, obstructing the migration of the metal ions in the AEM [95]. Ran [96] prepared an AEM by adding graphene oxide (GO) sheets with a high content of acid functional groups into imidazolium functionalized BPPO for HCl recovery from FeCl_2_–HCl solutions. The GO sheets act as auxiliary phases, which are key to overcome the trade-off effects. On the one hand, the GO sheets with high contents of –OH and –COOH groups could provide channels for the migration of H^+^. On the other hand, the GO sheets could act as a barrier for Fe^2+^ due to the interactions between Fe^2+^ and those groups on the GO sheets. Hence, the membrane showed high acid permeability (*U_H_* = 3 × 10^−2^ m/h) and selectivity (*S* = 200). In addition, although it was not mentioned in the paper, the GO sheets might obstruct the mobility of Fe^2+^ due to their large sizes and high valences to some extent.

Inspired by the above work, AEMs could be mixed with certain materials, such as metal organic frameworks or covalent organic frameworks, as both auxiliary materials for the migration of H^+^ and barriers for the mobility of metal ions to overcome the trade-off effects.

## 6. Membrane Stability

The excellent stability is another crucial parameter for application, as it determines the lifetime of the AEM [97]. Hence, researchers have made efforts to improve the stability of AEMs so that they can be used in DD for acid recovery for a long time.

### 6.1. Types of Functional Groups

The types of functional groups can influence the stability of AEMs, such as the quaternary ammonium groups used in AEMs, which result in inferior thermal and chemical stability. Mao [98] studied erosion effect for the AEM containing quaternized PPO. The results showed that the structure of this membrane was damaged mainly through the loss of quaternary ammonium groups. To overcome this deficiency of the quaternary ammonium groups, researchers modified the quaternary ammonium groups or replaced it with other alkaline functional groups in the AEMs.

**Figure 7:**
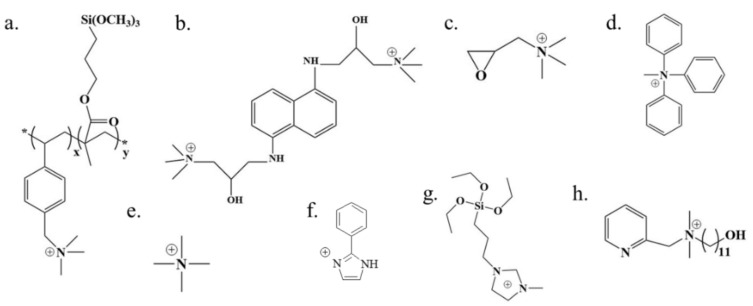
The structure of (**a**) quaternizated poly(VBC-co-γ-MPS), (**b**) Q-DAN, (**c**) glycidyl trimethyl ammonium chloride, (**d**) quaternary aromatic amine groups, (**e**) quaternary ammonium, (**f**) phenyl imidazole, (**g**) AESP (**h**) QUDAP [49,78,83,84,99,100,101,102].

On the one hand, the presence of aromatic groups on the quaternary ammonium groups could increase their mechanical and thermal stability. Wu [99] used vinylbenzyl chloride (VBC) as the monomer to prepare a quaternary multialkoxy silicon copolymer poly(VBC-co-*γ*-MPS) (Figure 7a), and then incorporated it into PVA to prepare a PVA-based AEM. Irfan [100] prepared a PVA-based AEM modified by quaternary 1,5-diaminonaphthalene (Q-DAN, Figure 7b). The presence of aromatic groups in poly(VBC-co-*γ*-MPS) and Q-DAN enhanced the thermal and mechanical stabilities of the AEM. Compared to the PVA-based AEM modified with glycidyl trimethyl ammonium chloride (Figure 7c) with a thermal degradation temperature (*T_d_*) of 218 °C [84], the PVA-based AEM modified with poly(VBC-co-*γ*-MPS) and Q-DAN showed higher thermal stabilities with *T_d_* values of 270 °C and 280 °C, respectively. Khan [49] prepared a PPO-based porous membrane modified with quaternary aromatic amine groups (Figure 7d). Compared to the PPO-based porous membrane functionalized with quaternary ammonium groups (Figure 7e) without aromatic groups (*T_d_* = 164.2 °C) [78], the membrane exhibited a higher thermal stability (thermal degradation temperature (*T_d_* = 179 °C)). Besides, Khan [101] prepared a series of PPO-based AEMs modified by phenylimidazole groups (Figure 7f). The results showed that these AEMs exhibited excellent acid stability.

On the other hand, using other alkaline functional groups instead of the quaternary ammonium groups can be used to overcome the problem of poor stability. Emmanuel [83] used 1-methyl imidazole to synthesize an anion exchange silica precursor (AESP, Figure 7g) and then prepared PVA-based membranes modified by AESP. The prepared membrane from AESP and PVA has excellent physicochemical stabilities compared to those of the quaternary ammonium group-based AEMs due to the imidazole rings. Besides, the presence of heterocyclic aromatic amines could improve the chemical and thermal stabilities of the prepared AEMs because they are more chemically and thermally stable than aliphatic amines. Irfan [102] incorporated quaternary 1-hydroxy-N,N-dimethyl-N-(pyridine-2-ylmethyl) methanaminium (QUDAP, Figure 7h) into PVA to prepare an AEM for HCl recovery. The AEM showed good flexibility owing to the long alkyl chain in QUDAP, which could remain slightly relaxed in the membrane matrix. The results showed that the thermal stability of the AEMs increased as QUDAP increased. In addition, the alkylation of hydrocarbons with long chains in heterocyclic aromatic amines could not only maintain stability but also enhance the flexibility of the membrane [82].

### 6.2. Crosslinking and Incorporating Inorganic Components

The ion exchange capacity and water uptake could be enhanced as the functionalization degree increased, which might result in severe swelling behavior [103]. 

**Figure 8:**
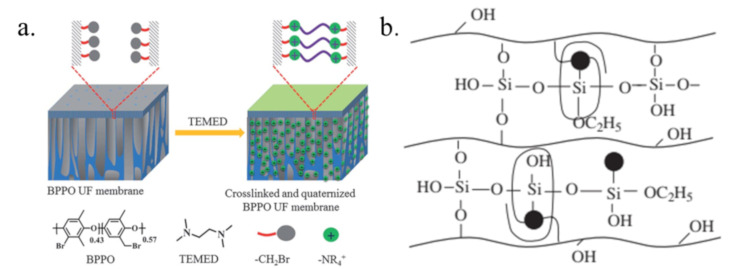
Schematic diagram of the preparation of crossliked and quaternized BPPO ultrafiltration membrane (**a**) and the structure of quaternized poly(2,6-dimethyl-1,4-phenylene oxide) (QPPO) and PVA-based membrane (**b**) [107,108].

One effective strategy for solving this problem and improving membrane stability is crosslinking [104,105]. Wu [106] studied the properties of PVA-based AEMs crosslinked by different crosslinking agents, such as small alkoxysilanes (tetraethoxysilane (TEOS), *γ*-glycidoxypropyltrimethoxysilane (GPTMS), monophenyltriethoxysilane (EPh)) and copolymers (glycidylmethacrylate (GMA) and methacryloxypropyl trimethoxy silane (MPS)). As a result, the AEM crosslinked by poly(GMA-co-MPS) with multiepoxy, alkoxy silicon (–Si(OCH_3_)_3_) groups and long chains demonstrated outstanding tensile properties and high stabilities. Lin [107] prepared a porous BPPO ultrafiltration AEM crosslinked and quaternized by N,N,N’,N’-tetramethylethylenediamine (TEMED), as shown in Figure 8a. The membrane showed no weight loss after immersion in hot acidic feed solution for 7 days and showed excellent thermal stability. In Wu’s work [108], the quaternized PPO and PVA-based AEM was crosslinked with double crosslinking agents, including monophenyl triethoxysilane (EPh) and tetraethoxysilane (TEOS), for HCl recovery (Figure 8b). The swelling behavior was generally restrained, and the thermal stability of the membrane increased as the crosslinking degree between the organic and inorganic phases increased.

Although the AEMs with a high crosslinking degree show excellent stability, their compact structure, and the loss of functional groups due to crosslinking can reduce the acid permeability. Fortunately, there are other approaches used to improve stability, such as introducing inorganic materials into the membrane. Sharma [109] prepared PVA-based AEMs with different contents of functionalized graphene nanoribbons (f-GNRs) for HCl recovery from FeCl_2_–HCl solutions. The results showed that the membrane exhibited excellent stability and less swelling because f-GNR not only exhibited a high stability but could also be considered to be a filler for the polymer matrix and reduce its free volume, which resulted in a denser membrane. In addition, the acid permeabilities increased by the presence of f-GNRs in the AEMs and the dialysis coefficient is 53 × 10^−3^ m/h when the concentration of the f-GNR is 0.1 wt.%.

## 7. The Integration of Diffusion Dialysis with Other Technologies

The DD process still shows a limited processing capability for acid recovery due to the restriction of the equilibrium concentration. Hence, it is necessary to integrate diffusion dialysis with other technologies such as pressure, electric fields and continuous processes to overcome the limitation and improve the efficiency of the acid recovery [26,110,111].

### 7.1. The Integration of Diffusion Dialysis with Pressure

In the DD process, the acid concentration achieved in the receiving solution is too low for reuse. Furthermore, water might be transported from the receiving side to the feed side due to the osmotic pressure difference between the sides, which reduces the concentration on the feed side, resulting in a driving force for the lower mass transport. Hence, using pressure as the auxiliary to drive the migration of acid in the DD process could achieve a higher acid concentration in the receiving side and prevent water osmosis [112]. Yun [113] used thermally cross-linked branched polyethyleneimine (b-PEI) with strong positive charges to coat the surface of a polyethersulfone (PES) nanofiltration membrane for the recovery of HCl from MgCl_2_, MgSO_4_, NaCl or Na_2_SO_4_ solution. The results showed that the membrane exhibited excellent rejection of metal ions, especially Mg^2+^ (95%) at 30 bars of pressure. Furthermore, the membrane showed good stability and could maintain selective acid permeability for a month. Zhang [114] proposed a pressure-concentration double-driven DD process to recover H_2_SO_4_ from FeSO_4_-H_2_SO_4_ solution using a commercial DF-120 membrane. As a result, the dialysis coefficient of H^+^ increased from 12 × 10^−4^ to 39 × 10^−4^ m/h as the pressure ranged from 0 to 0.08 MPa, while the acid selectivity remained at an acceptable value (*S* ≈ 65). Moreover, the use of pressure could prevent osmosis from the receiving side to the feed side.

In summary, pressure-assisted DD uses an additional driving force to enhance the acid recovery performance in practical applications, especially for feed solutions with low acid concentrations.

### 7.2. The Integration of Diffusion Dialysis with an Electric Field

**Figure 9:**
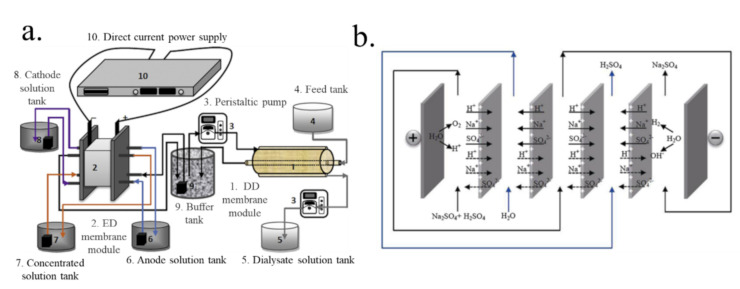
Flow chart of experimental apparatus (**a**). The schematic representation of the ED assisted DD stack (**b**) [118,119].

Using an electric field as an extra driving force in DD could overcome the disadvantage of DD. One method is integrating electrodialysis (ED) and DD to recover acid. As a common membrane technology-based ion exchange membrane, ED is widely used in the separation of ions driven by electrical fields [115,116,117]. Zhang [118] integrated DD and ED to recover HCl from simulated chemosynthesis aluminum foil wastewater, as shown in Figure 9a. The results showed that the integration of ED and DD was a more effective method to recover HCl than DD alone. This is because the integration could not only save water but also recover high purity HCl, which could be directly reused. In addition, Zhang [119] used a weak electric field as a secondary driving force in DD to improve the performance of H_2_SO_4_ recovery from Na_2_SO_4_–H_2_SO_4_ solutions using the DF-120 membrane (Figure 9b). There were one or many repeating AEMs in the weak-electric-field-assisted DD stack, which was different from the ED stack with cation-and-anion exchange membranes. In addition, the acid permeability in the weak-electric-field-assisted DD process was higher than that in DD alone, and the energy consumption was relatively low.

### 7.3. The Integration of Diffusion Dialysis with a Continuous Process

The reported acid recoveries using AEMs in DD were mainly obtained in a batch dialyzer. However, there is limited capacity for acid recovery in the batch dialyzer due to the limited membrane area for the migration of acid in practical applications [120]. Compared to the batch dialyzer, a continuous dialyzer for acid recovery exhibits various advantages, such as higher productivity, smaller sized dialysis equipment, lower costs and easier operation, and continuous processes could be appropriate for practical production [33,121,122]. 

**Figure 10:**
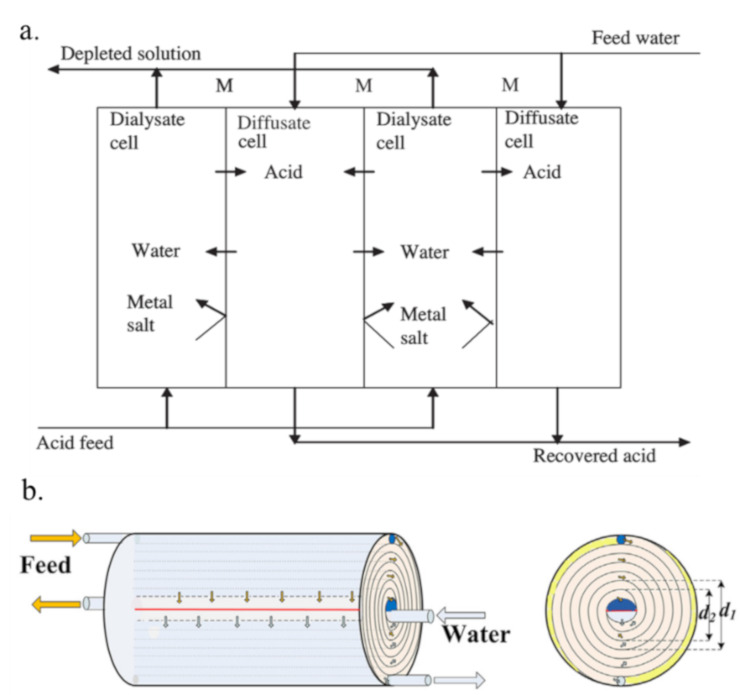
Scheme of Plate-and-frame diffusion dialysis (PFDD) module (**a**) and spiral wound diffusion dialysis (SWDD) module (**b**) [123,128].

The plate-and-frame diffusion dialysis (PFDD, Figure 10a) module is one kind of continuous dialyzers which is comprised of a series of flat membrane sheets to enhance the effective areas for acid recovery [123]. Li [124] recovered H_2_SO_4_ from acid leaching solution (containing metal ions such as V, Al and Fe ions) using a PFDD module with the commercial DF-120 membrane. As a result, the recovery ratio of H_2_SO_4_ and the rejection of V, Al and Fe ions reached 84 wt.%, 93 wt.%, 92 wt.%, and 85 wt.%, respectively, at a flow rate of 2.1 × 10^−3^ m^3^/h m^2^ and flow rate ratio of water to feed of 1.1–1.3 at 25 °C. The flow rate and water flow rate ratio are important parameters which influence the transport efficiency of acid diffusion process. Generally, with the increasing of the flow rate, the acid recovery efficiencies increase and get a maximum initially, and then decrease [123,124]. At a low flow rate of the feed solution, the processing capacity is low, and the water reverse osmosis phenomenon is enhanced duo to the concentration polarization. However, a high flow rate is also not beneficial to the acid recovery efficiency, due to the short retention time for proton migration. In this situation, the time is not sufficient and only small parts of feed element could permeate the membrane, resulting in a decrease for the acid recovery ratio [125,126]. Hence, it is necessary to find an appropriate flow rate to get a high acid recovery ratio. For the flow rate ratio of water to feed, the acid recovery usually increases when the water flow rate ratio increases. However, this increase is not linear at higher water flow rate ratios because of the damage of the diffusion boundary layers between the membrane and the solution interface [21]. Besides, the metal ions rejection usually decreases at high water flow rate ratios [123]. Hence, it is also essential to optimize the water flow rate ratio to obtain a high efficiency recovery for acid using DD processes. Kim [123] also studied the recovery of H_3_PO_4_ from a mixed acid solution (containing Al ions) using a PFDD module that contained four diffusate cells divided by three AEMs. In this work, 85 wt.% H_3_PO_4_ could be recovered by DD, while 3.68 mg/kg of Al ions could leak into the diffusate cell. In addition, some mathematical models have been developed to describe the performance of continuous DD with AEMs for acid recovery. In Palatý’s work [127], the recovery of H_2_SO_4_ from a H_2_SO_4_-Na_2_SO_4_ mixture was studied in a two-cell counter current dialyzer equipped with a commercial Neosepta-AFN membrane at steady operation. Then, a rigorous mathematical model was developed to describe both the convective transport in the cells and the transport of H_2_SO_4_ and Na_2_SO_4_ through the AEM and liquid films.

However, the PFDD module has some disadvantages, including a complicated assembly process, bulky equipment, and limited mass transfer [128]. Another type of module, a spiral wound diffusion dialysis (SWDD, Figure 10b) module, which uses a long, flat membrane piece curled into a spiral together with partitions in the DD process for acid recovery. The SWDD module exhibits merits such as a smaller equipment size, relatively higher acid recovery and convenient transportation [128]. Zhang [126] recovered HCl from a HCl–AlCl_3_ solution using a SWDD module with DF-120. Compared to the PFDD module, the SWDD module exhibited a relatively high acid recovery ratio (84.3 wt.%), low Al ion leakage ratio (less than 4 wt.%) and a similar time to reach equilibrium (3 h). However, the SWDD module is difficult to disassemble, check and replace due to the sealing of the two sides of the AEM with glue to prevent the leakage of the solution [129].

**Figure 11:**
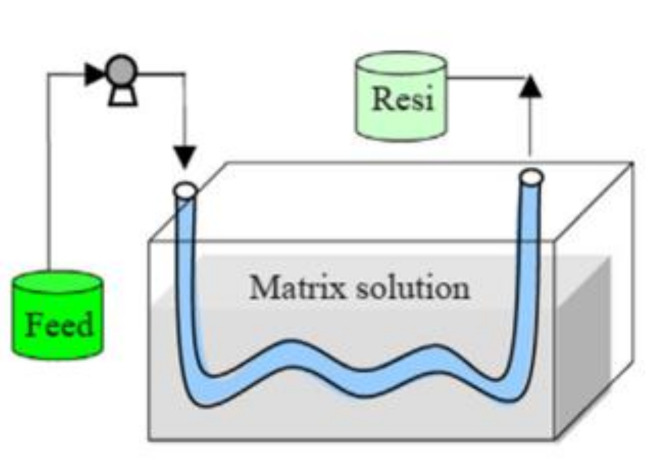
The schematic diagram for the static semi-continuous process using the PVA-based tubular membrane (“Resi” means residual liquor)[129].

Hence, inspired by “blood vessels” from a biological perspective, another module comprising tubular AEMs immersed in the solution was designed for acid recovery in DD. The hollow-fiber AEM was synthesized from BPPO and 1-methyl-2-pyrrolidone (NMP) by Xu and then aminated by dimethylethanolamine (DMEA) and trimethylamine [130]. Considering that the hollow-fiber-type dialyzers are more effective than the PFDD and SWDD modules, the hollow-fiber AEM could have broad applications. In Ye’s work [129], a PVA-based tubular membrane was prepared to recover HCl in semicontinuous (Figure 11). The semicontinuous process was performed by immersing the membrane into the static matrix solution (water) and flowing the feed solution through the membrane. The semicontinuous process exhibited energy savings, easy operation, and a high feed capacity with a high acid recovery ratio of 71.1–75.5 wt.%. The recovered HCl concentration and acid recovery ratio in the continuous process were 1.27 mol/L and 65.2 wt.%, respectively, which could be on par with those in the PFDD process with recovered acid concentrations of 0.54–1.01 mol/L and recovery ratios of 29.2–80.9 wt.%. However, the new module with a tubular membrane has not been fully studied and has some drawbacks in its preparation, but it could provide a novel strategy for recovering acid in practical applications.

## 8. Summary and Perspective

Diffusion dialysis (DD), which offers low energy consumption, easy operation and environmentally friendliness, was comprehensively applied in the area of acid recovery from acidic waste solutions. An anion exchange membrane (AEM) for DD with excellent properties, including acid permeability, acid selectivity and membrane stability, is an important factor that determines the efficiency and processing capacity of the acid recovery. Substantial effort was devoted by researchers to developing three main methods to improve the properties of AEMs: functionalizing the membrane, changing the structure of the membrane, adding other materials and/or crosslinking. Besides, the works on diminishing the trade-off effects of AEMs between acid permeability and selectivity and integration DD using AEMs with other technologies were made for acid recovery. By reviewing the many reported works, we propose the following problems and directions for further improvements in AEMs: (1) Chemicals with high stability and alkalinity can be used as modifiers to prepare AEMs with improved acid recovery and stability. (2) Materials with a size-sieving effect could be introduced into AEMs to enhance acid selectivity. (3) Acidic functional groups, such as –COOH and –HSO_3_, have an excellent effect on the acid recovery of AEMs and could even overcome trade-off effects. Further research should be conducted to broaden the kinds of acid-alkali ion pair functional groups used in AEMs. In addition, the mechanism and interactions during the migration of ions in the AEM should be clearly explained using calculations and simulations. Besides, integrating with various technologies, such as pressure, an electric field, and a continuous process, into DD processes could enhance its processing capacity, which enhances its potential for industrial applications. Furthermore, comprehensive optimization of the DD process using AEMs for acid recovery from acidic waste solutions should consider the operational costs, product quality and so on.

## Figures and Tables

**Table 1:** A summary of methods for acid recovery.

Methods	Description	Advantages	Disadvantages
Crystallization [3,4]	The solubility of saline, such as FeCl_2_ or AlCl_3_, in the waste solutions reduces at low temperature, resulting in crystallization and separation.	Less investment in equipment simple to install	Long production period Low processing capacity High energy cost
Solvent extraction [5,6,7]	Extraction agents are used to extract acid or metal ions selectively from waste solutions. Then, the acid or metal ions could be collected via back-extraction.	High yield and selectivity Pure product	Complicated operation Bad for environmental owing to the extraction agents.
Ion exchange resin [8,9,10]	Ion exchange materials are used to absorb acids or metal ions in the waste solution, and then the acids or metal ions could be desorbed from the solid phases.	High selectivity Simple to operation	High costs Low adsorption capacities
Membrane technology [13,18,19]	Membrane technologies contain the reverse osmosis process, electrodialysis and diffusion dialysis which correspond to pressure, an electric field or activity as driving forces, respectively. Acids are transported through membranes from feed side to the receiving side under the driving forces.	High efficiency Reliable Simple to install and scale up.	Limited processing capacities

**Table 2:** The economic investment of the DD system using AEMs for acid recovery [13,22,33,34].

Material	Price ($)
Diffusion dialysis unit	170,000–1,350,000
Membranes replacement	15,000–300,000
Auxiliary (pump, circuit, valve, and tank)	150,000
Power, labor, and others	3000–5000
Total	338,000–1,805,000
Write-off (investment-recovery period): 4.8–26.4 months	

**Table 3:** The comparison of the comparison of dialysis coefficient (*U_H_*) and selectivity (*S*) for H^+^ over metal ions of the reported membranes at 25 °C.

Membrane	Structure	*U_H_* (10^−3^ m/h)	*S*	Simulated Solution System
Commercial Neosepta-AFX [85]	Dense	4	25	0.05 M FeCl_3_–2 M H_2_SO_4_
Commercial DF-120 [44]	Dense	4	19	0.25 M FeCl_2_–1.0 M HCl
Neosepta-AFX modified with 5 vol.% pyrrole [85]	Dense	4	48	0.05 M FeCl_3_–2 M H_2_SO_4_
BPPO-based AEM crosslinked with a multi-amine oligomer [87]	Dense	9	2074	0.59 M FeSO_4_–1.03 M H_2_SO_4_
The pore-filled AEM with PE and polypyrrole [74]	Porous	10–11	36–54	0.05 M FeCl_3_–2 M H_2_SO_4_
The quaternized BPPO AEM [57]	Dense	3–13	H/Fe: 40	0.15 M TiO_2_–0.17 M FeSO_4_–0.25 M H_2_SO_4_
			H/Ti: 70	
The PPO-based AEM with quaternized nitrogen and –COOH groups [64]	Dense	5–19	73–390	0.25 M FeCl_2_–1.0 M HCl
The PVA-based AEM modified by pyridinium [82]	Dense	17–25	31–58	0.25 M FeCl_2_–1.0 M HCl
The BPPO-based AEM with sponge-like pores [86]	porous	15–20	81–665	0.21 M FeCl_2_–1 M HCl
		22–28	100–2033	0.46 M AlCl_3_–2.12 M HCl
The PECs/PVA AEM [44]	Dense	3–23	40–90	0.25 M FeCl_2_–1.0 M HCl
The PPO-based ultrafiltration AEM containing –COOH groups and quaternary ammonium [78]	Porous	20–25	28–46	0.25 M FeCl_2_–1.0 M HCl
The PVC-based AEM immobilized by DMAM and DVB [65]	Dense	12–40	36–61	0.18 M FeCl_2_–0.81 M HCl
The nanofiber AEM [80]	Porous	41	50	0.225 M FeCl_2_–1 M HCl
PANI-based AEM [56]	Porous	32	20	5% FeCl_3_–3.5 M HCl ^-^
A-PANI-based AEM [56]	Porous	42	17	5% FeCl_3_–3.5 M HCl ^-^
The PVA-based AEM modified by multisilicon copolymers [63]	Dense	10–43	22–39	0.12 M FeCl_2_–1 M HCl
The double quaternization PVA-based membrane [67]	Dense	30–45	21–32	0.25 M FeCl_2_–1.0 M HCl
ESM/PSF membrane [79]	Porous	10–46	33–93	0.125 M FeCl_2_–0.5 M HCl
NP/PSF membrane [68]	Porous	47	154	0.125 M FeCl_2_–0.5 M HCl
Imidazolium functionalized PVA-based AEM [83].	Dense	19–48	13–53	0.25 M FeCl_2_–1.0 M HCl
The BPPO-based AEM modified pyrrolidinium [50]	Dense	18–49	36–66	0.18 M FeCl_2_–0.81 M HCl
PVA-based AEMs by grafting different contents of allyltrimethylammonium chloride [66].	Dense	17–60	8–26	0.18 M FeCl_2_–0.81 M HCl
The PSF-based AEM [47]	Porous	65	34	0.2 M FeCl_2_–1 M HCl
The BPPO-based AEM modified MP [61]	Dense	11–66	25–78	0.25 M FeCl_2_–1 M HCl
The PPO-based ultrafiltration AME modified by PEI and TMA [77]	Porous	56–70	11–21	0.2 M FeCl_2_–1 M HCl

## Appendix A. List of Abbreviations

A-PANI	Poly(o-anisidine)
b-PEI	Branched polyethyleneimine
BPPO	Brominated poly(2,6-dimethyl-1,4-phenylene oxide)
DABVO	1,4-diazabicyclo[2,2,2]octane
DMAM	Dimethylaminoethyl methacrylate
DMEA	Dimethylethanolamine
DVB	Divinylbenzene
GMA	Glycidylmethacrylate
GO	Graphene oxide
NMP	1-methyl-2-pyrrolidone
MPS	Methacryloxypropyl trimethoxy silane
PANI	Polyaniline
PE	Polyethylene
PEI	Polyethyleneimine
PES	Polyethersulfone
PFDD	Plate-and-frame diffusion dialysis
PP	Polypropylene
PPO	Poly(2,6-dimethyl-1,4-phenylene oxide)
PSF	Polysulfone
PVA	Polyvinyl alcohol
PVC	Polyvinyl chloride
QBAPB	4,4′-(1,1′-biphenyl-4,4′-diyldioxy)dianiline
Q-DAN	Quaternary 1,5-diaminonaphthalene
QUDAP	Quaternary 1-hydroxy-N,N-dimethyl-N-(pyridine-2-ylmethyl) methanaminium
SWDD	Spiral wound diffusion dialysis
TDA	Tris(2-(2-methoxyethoxy)ethyl)amine
TEMED	N,N,N′,N′-tetramethylethylenediamine
TMPDA	N,N,N′,N′-tetramethyl-1,3-propanediamine
